# Seizure freedom after surgical resection of diffusion‐weighted magnetic resonance imaging abnormalities

**DOI:** 10.1111/epi.18490

**Published:** 2025-06-10

**Authors:** Jonathan Horsley, Gerard Hall, Callum Simpson, Csaba Kozma, Rhys Thomas, Yujiang Wang, Jane de Tisi, Anna Miserocchi, Andrew McEvoy, Sjoerd Vos, Gavin Winston, John Duncan, Peter N. Taylor

**Affiliations:** ^1^ CNNP Lab, Interdisciplinary Computing and Complex BioSystems Group, School of Computing Newcastle University Newcastle Upon Tyne UK; ^2^ Translational and Clinical Research Institute, Faculty of Medical Sciences Newcastle University Newcastle Upon Tyne UK; ^3^ Department of Clinical and Experimental Epilepsy UCL Queen Square Institute of Neurology, University College London London UK; ^4^ Centre for Microscopy, Characterisation, and Analysis University of Western Australia Nedlands Western Australia Australia; ^5^ Centre for Medical Image Computing, Computer Science Department University College London London UK; ^6^ Division of Neurology, Department of Medicine Queen's University Kingston Ontario Canada

**Keywords:** computational, epilepsy, neuroimaging, surgery, treatment

## Abstract

**Objective:**

Successful epilepsy surgery requires accurate localization and removal of the epileptogenic zone. Neuroimaging helps detect structural brain abnormalities to guide surgery, but current clinical practice does not use diffusion‐weighted magnetic resonance imaging (dwMRI). However, previous work has shown that diffusion abnormalities are present in epilepsy and may relate to the epileptogenic zone. Here, we investigate whether surgical resection of diffusion abnormalities relates to postoperative seizure freedom.

**Methods:**

We investigated the association between surgical resection of diffusion abnormalities and postoperative seizure freedom in 200 individuals with drug‐resistant focal epilepsy using dwMRI. A cohort of 97 healthy controls provided a normative baseline for dwMRI metrics, allowing calculation of voxelwise *z*‐scores to identify abnormal clusters in both gray and white matter.

**Results:**

Surgical resections overlapping with the largest abnormal cluster significantly correlated with sustained seizure freedom at 12 months (83% vs. 55%; p<.0001) and over 5 years (p<.0001). Notably, resecting only a small proportion of the largest cluster was associated with better seizure outcomes than cases with no resection of this cluster (p=.008). Furthermore, sparing the largest cluster but resecting other large clusters still improved seizure freedom rates compared to no overlap (p=.03).

**Significance:**

Our results suggest that abnormal clusters, identified using dwMRI, are integral to the epileptogenic network, and even a partial removal of such an abnormal cluster is sufficient to achieve seizure freedom. This study highlights the potential of incorporating dwMRI into presurgical planning to improve outcomes in focal epilepsy by reliably identifying and targeting diffusion abnormalities.

**Key points**

- Removal (or disruption) of the epileptogenic zone is required for seizure freedom following epilepsy surgery.
- dwMRI is not currently part of standard presurgical planning to identify epileptogenic brain regions.
- Resection of diffusion abnormalities detected on dwMRI was strongly associated with seizure freedom, suggesting these abnormalities may help pinpoint the epileptogenic zone.

## 1 INTRODUCTION

Up to half of people who undergo resective surgery for epilepsy continue to have seizures in the long term.^1^ Current clinical approaches to determine which region(s) of the brain to remove in surgery involve the qualitative assessment of a variety of structural and functional data, including seizure semiology, structural magnetic resonance imaging (MRI), electroencephalography, functional MRI, magnetoencephalography, fluorodeoxyglucose positron emission tomography, and single photon emission computed tomography.^2^ These data are used to infer the location of the epileptogenic zone (EZ)—the area of the brain necessary for the generation of epileptic seizures.^3^ By definition, in those people who continue to have seizures after surgery, the EZ was not sufficiently disrupted by the surgery, possibly due to mislocalization. Successful surgery therefore requires accurate localization and disruption of the EZ, and new data sources are needed to assist with this localization in the clinic.

Studies have shown that people with epilepsy have abnormalities detectable by diffusion‐weighted MRI (dwMRI).^4^ Despite the magnitude of dwMRI abnormalities being larger closer to the suspected EZ in both temporal^4^, ^5^ and extratemporal^6^ epilepsies, presurgical evaluations do not typically use dwMRI for localization of the EZ. However, dwMRI may often be acquired to map white matter tracts and avoid postoperative neurological deficits caused by surgery.^2^ Because dwMRI is already acquired in many cases, it may provide additional benefit to existing presurgical evaluations at little extra cost, if it is shown to be able to localize the EZ.

It is currently not fully understood whether dwMRI abnormalities represent the EZ or some wider consequence of epileptic seizures. Previous work used dwMRI data to predict postsurgical outcomes, but often at the group level,^7^, ^8^ with small sample sizes,^7^, ^9^, ^10^, ^11^, ^12^ or not validated using resection masks and postsurgical outcomes.^13^, ^14^ Additionally, many of these studies conducted analyses at the spatial scale of entire tracts or structural connectomes. White matter diffusion abnormalities, however, are not spread evenly along an entire tract, but may be localized to specific segments,^8^ and resection of these abnormal segments may be associated with seizure freedom, at least in specific tracts and types of epilepsy.^8^ To improve clinical utility of dwMRI abnormalities, there is a need to (1) identify localized abnormalities in individual subjects and (2) determine whether resection of these abnormalities is associated with improved postsurgical outcomes.

In this study, we identified dwMRI abnormalities at the voxel level in 200 surgically treated people with drug‐resistant focal epilepsy. We then assessed whether the location of these abnormalities were resected and analyzed the extent to which resection of dwMRI abnormalities resulted in seizure freedom up to 5 years after surgery.

## 2 MATERIALS AND METHODS

### 2.1 Subjects

We retrospectively studied 200 individuals with surgically treated drug‐resistant focal epilepsy from the National Hospital of Neurology and Neurosurgery, London, UK. Analysis of pseudoanonymized data from the University College London Hospitals (UCLH) Epilepsy Surgery Database was approved by the Health Research Authority (22/SC/016).

Table 1 summarizes the subjects in this cohort, stratified by postsurgical seizure freedom. Data from 97 healthy controls formed a normative comparison group and were age‐ and sex‐matched to the individuals with epilepsy (individuals with epilepsy: mean age = 36.6 ± 11.3 years, 56% female; controls: mean age = 39.6 ± 12.9 years, 61% female). Of the 200 individuals with epilepsy, 83 had histopathological evidence of hippocampal sclerosis, 26 had focal cortical dysplasia, 25 had dysembryoplastic neuroepithelial tumor, 14 had cavernoma, 12 had dual pathology, two had glioma, and one had treble pathology. The remaining 37 had some other unspecified pathology. The most common type of surgical resection was temporal lobectomy (*n* = 140), followed by frontal lobectomy (*n* = 28), temporal lesionectomy (*n* = 15), frontal lesionectomy (*n* = 7), parietal lesionectomy (*n* = 4), occipital lobectomy (*n* = 3), parietal lobectomy (*n* = 1), occipitoparietal lobectomy (*n* = 1), and temporo‐occipital lesionectomy (*n* = 1).

**TABLE 1:** Patient data by 12‐month postsurgical seizure freedom.

Characteristic	ILAE 1, 2	ILAE 3+	Test statistic
*n*	139	61	
Onset age, years, median (IQR)	12 (14.5)	15 (13.0)	W=3617, p=.10
Sex, male:female	56:83	33:28	$\chi^{2}=2.74$, p=.10
Type, temporal:extratemporal	108:31	47:14	$\chi^{2}\approx .00$, p=1
Side, left:right	80:59	24:37	$\chi^{2}=4.93$, p=.03
MRI, nonlesional:lesional	18:121	14:47	$\chi^{2}=2.45$, p=.12

*Note*: The difference in onset age between groups was assessed using a Wilcoxon rank‐sum test. Other differences between groups were assessed using chi‐squared tests.

Abbreviations: ILAE, International League Against Epilepsy; IQR, interquartile range; MRI, magnetic resonance imaging.

### 2.2 Data acquisition

dwMRI acquisition were obtained in two separate cohorts using different scanning protocols. The first cohort was collected between 2009 and 2013 and had 107 patients and 29 controls. The second cohort was collected between 2014 and 2019 and comprised 93 patients and 68 controls.

The first cohort of dwMRI data used a cardiac‐triggered single‐shot spin‐echo planar imaging sequence^15^ with echo time = 73 ms. Sixty contiguous 2.4‐mm‐thick axial slices were obtained covering the whole brain, with diffusion sensitizing gradients applied in each of 52 noncollinear directions (b‐value of 1200 $s/mm^{2}$ [δ=21ms, Δ=29ms, using full gradient strength of 40 $\mathrm{mT}m^{-1}$]) along with six non‐diffusion‐weighted scans. The gradient directions were calculated and ordered as described elsewhere.^16^ The field of view was 24 × 24 $cm^{2}$, and the acquisition matrix size was 96 × 96, zero filled to 128 × 128 during reconstruction, giving a reconstructed voxel size of 1.875 × 1.875 × 2.4 mm^3^.

The second cohort of dwMRI data were acquired using a single‐shot spin‐echo planar imaging sequence with echo time = 74.1 ms. Seventy contiguous 2‐mm‐thick axial slices were obtained covering the whole brain. A total of 115 volumes were acquired with 11, eight, 32, and 64 gradient directions at b‐values of 0, 300, 700, and 2500 $s/mm^{2}$, respectively (δ=21.5ms, Δ=35.9ms) as well as a single *b* = 0 image with reverse phase encoding (B0). The field of view was 25.6 × 25.6 $cm^{2}$, and the acquisition matrix size was 128 × 128, giving a reconstructed voxel size of 2 × 2 × 2 mm^3^.

### 2.3 Data processing and registration

The dwMRI scans from both cohorts were processed identically. The scans were denoised,^17^ Gibbs‐unringed,^18^ and corrected for signal drift.^19^ Furthermore, because one cohort did not have reverse phase‐encoded B0s, the Synb0‐DisCo^20^, ^21^ tool was used to create a nondistorted synthetic image from each participant's corresponding T1 structural MRI. The Synb0‐DisCo tool was run for both cohorts irrespective of the existence of reverse phase‐encoded images to ensure continuity between the processing of the two cohorts. The calculated nondistorted synthetic image was subsequently input into TOPUP^22^, ^23^ and EDDY^24^ to correct for warping, eddy current‐induced distortions, and motion. Lastly, correction for signal bias was applied using N4 bias field correction.^25^

**FIGURE 1:**
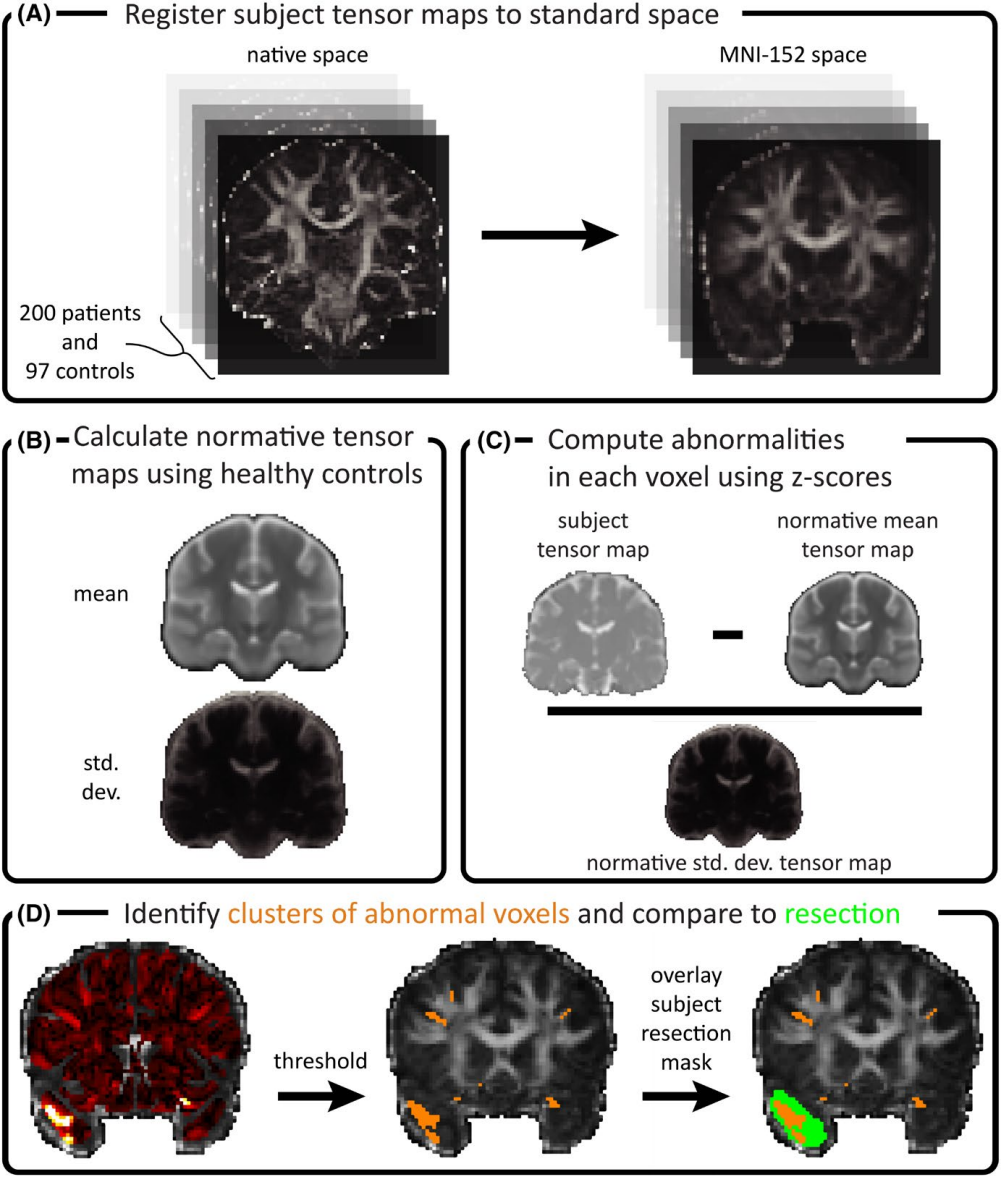
Abnormality calculation pipeline. (A) All subjects were registered to the same MNI‐152 standard space. (B) Normative tensor maps of diffusion were calculated for mean diffusivity (MD), axial diffusivity, radial diffusivity, and fractional anisotropy by calculating the mean and SD of the tensor values across healthy controls in each voxel (shown here for MD only). (C) Abnormality values were calculated by *z*‐scoring each subjects' tensor values against the same voxel in healthy controls. (D) Abnormalities were thresholded at *z* = 3 and compared to the resection mask for each subject.

After preprocessing, tensor maps were calculated using FSL's DTIFIT tool, and the fractional anisotropy (FA) maps from each individual were registered to a standard template (HCP_10065_FA) in MNI‐152 standard space (Figure 1A). All b‐values were used in tensor reconstruction for both cohorts. Registration used the *antsRegistrationSyN.sh* script from ANTs, which employed both linear (affine) and nonlinear (diffeomorphic SyN) transformations.^26^, ^27^ Using the transformation files calculated from the prior registration, all tensor maps (FA, mean diffusivity [MD], axial diffusivity [AD], and radial diffusivity [RD]) were then moved into standard space using the *antsApplyTransforms* tool with a trilinear interpolation. No other smoothing was applied.

### 2.4 Abnormality calculation

All analysis was performed in R version 4.3.0.

Normative diffusion tensor maps were created using healthy controls. We computed both the mean and SD of FA, MD, AD, and RD in each voxel across controls (Figure 1B). Separate normative maps were created for both cohorts. Voxels in cerebrospinal fluid, skull, brain stem, and cerebellum were discounted. These normative maps acted as a healthy baseline, against which we assessed individual subjects.

We calculated abnormalities in each voxel by *z*‐scoring against the corresponding voxel in the normative map of the same cohort (Figure 1C). This *z*‐scoring was done separately for FA, MD, AD, and RD. In each case, the abnormality values in each voxel specified the number of SDs away from the healthy mean. Abnormalities were calculated independently for each voxel, but abnormalities in neighboring voxels may be more indicative of a true abnormal signal rather than noise. To boost the signal‐to‐noise ratio by considering neighboring abnormalities, we applied probabilistic threshold free clustering enhancement (pTFCE).^28^

Abnormal voxels were defined as those exceeding a (pTFCE‐enhanced) *z*‐score of 3, and clusters of neighboring abnormal voxels were identified (Figure 1D). Supplementary Analysis S1 shows results for alternative thresholds. Clusters were ordered by volume, and substantially large clusters were detected. Specifically, we defined substantially large clusters in each individual as those that exceeded a volume threshold that differentiated them from smaller, potentially spurious clusters. This threshold was derived in a data‐driven manner using change‐point analysis^29^ with a gamma prior distribution.

### 2.5 Resection mask generation

Resection masks were generated using a semiautomated approach as described previously.^30^ Briefly, we used postoperative imaging to generate masks of the tissue that was subsequently resected. Masks were initially generated automatically using a custom‐built software pipeline, using FastSurfer,^31^ ANTs,^26^ and ATROPOS.^32^ These automated masks were visually inspected and, if needed, manually corrected to ensure quality. The resection masks were then registered to the same standard (MNI‐152) space as the abnormality maps.

Within each subject, cluster abnormality maps were overlaid with the resection mask. From this, we calculated the proportion of the largest cluster resected. The same proportion was calculated for all substantially large clusters within a subject. We compared these cluster resection proportions to the likelihood of a person remaining free from disabling seizures following surgery. We hypothesized that resection of the largest or other substantially large clusters would be associated with postoperative seizure freedom.

## 3 RESULTS

Clusters of diffusion abnormalities were calculated in each subject for MD, which we will present in the following main text. Previous work suggested that the location of MD abnormalities was more predictive of postsurgical outcome than FA.^8^ Alternative measures (AD, RD, and FA) for abnormality cluster analyses are presented in Supplementary Analysis S2 for completeness, and show broadly similar results.

### 3.1 Resection of the largest cluster is associated with good outcome

First, we investigated whether resection of the largest cluster was associated with postsurgical seizure freedom using survival analysis. Within an individual, the largest cluster was defined as resected if there was any overlap with the resection mask. At each yearly follow‐up, a person was defined as seizure‐free if they had no debilitating seizures (i.e., International League Against Epilepsy 1 or 2); otherwise, they were defined as not seizure‐free.

**FIGURE 2:**
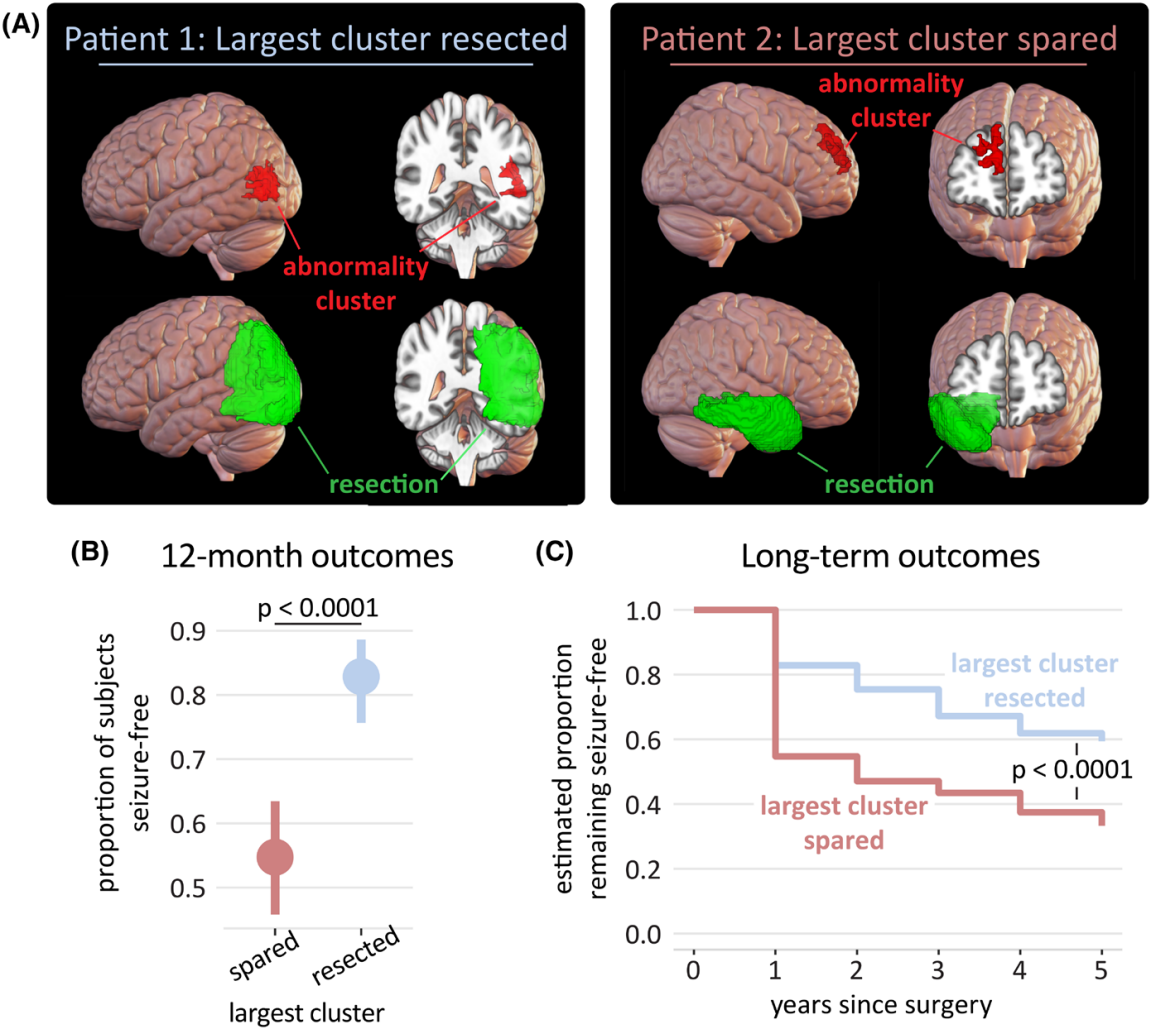
Resection of the largest cluster is associated with good outcome. (A) The largest cluster of (mean diffusivity) diffusion abnormalities are shown in red for two example subjects. Both subject were clinically assessed as magnetic resonance imaging‐negative, that is, nonlesional. In the left‐hand subpanel (Patient 1), the resection mask (green) overlapped with the largest cluster, and this subject was seizure‐free (International League Against Epilepsy [ILAE] 1) at 12 months postsurgery. In the right‐hand subpanel (Patient 2), the resection mask (green) did not overlap with the largest cluster and this subject was not seizure‐free (ILAE 5) for the 4 years of available follow‐up data. (B, C) Across the cohort, overlap between the largest cluster and the resection mask was associated with a significantly improved rate of seizure freedom (B) at 12 months and (C) over 5 years postsurgery. Error bars indicate 90% confidence intervals.

After 12 months, the seizure freedom rate among those with the largest cluster resected was 83%, compared to 55% among those with the largest cluster spared (p<.0001; Figure 2). Resecting the largest cluster significantly predicted outcome in the long (5 years) term (p<.0001) and was more predictive than a range of clinical features (Supplementary Analysis S3). Sample sizes at each year of follow‐up are presented in Table S1. Subgroup analysis, albeit with reduced statistical power to detect significant effects, observed similar trends in both MRI‐negative (n=32, p=.07) and MRI‐positive cohorts (n=168, p=.002; Supplementary Analysis S4), in both scanning protocol cohorts (Supplementary Analysis S5) in both temporal lobe epilepsy and extra‐temporal lobe epilepsy cohorts (Supplementary Analysis S6) and in both left‐ and right‐sided resection cohorts (Supplementary Analysis S7). The size of resection did not predict outcome (Supplementary Analysis S8).

### 3.2 Resection of even a small proportion of the largest cluster can still lead to a good outcome

**FIGURE 3:**
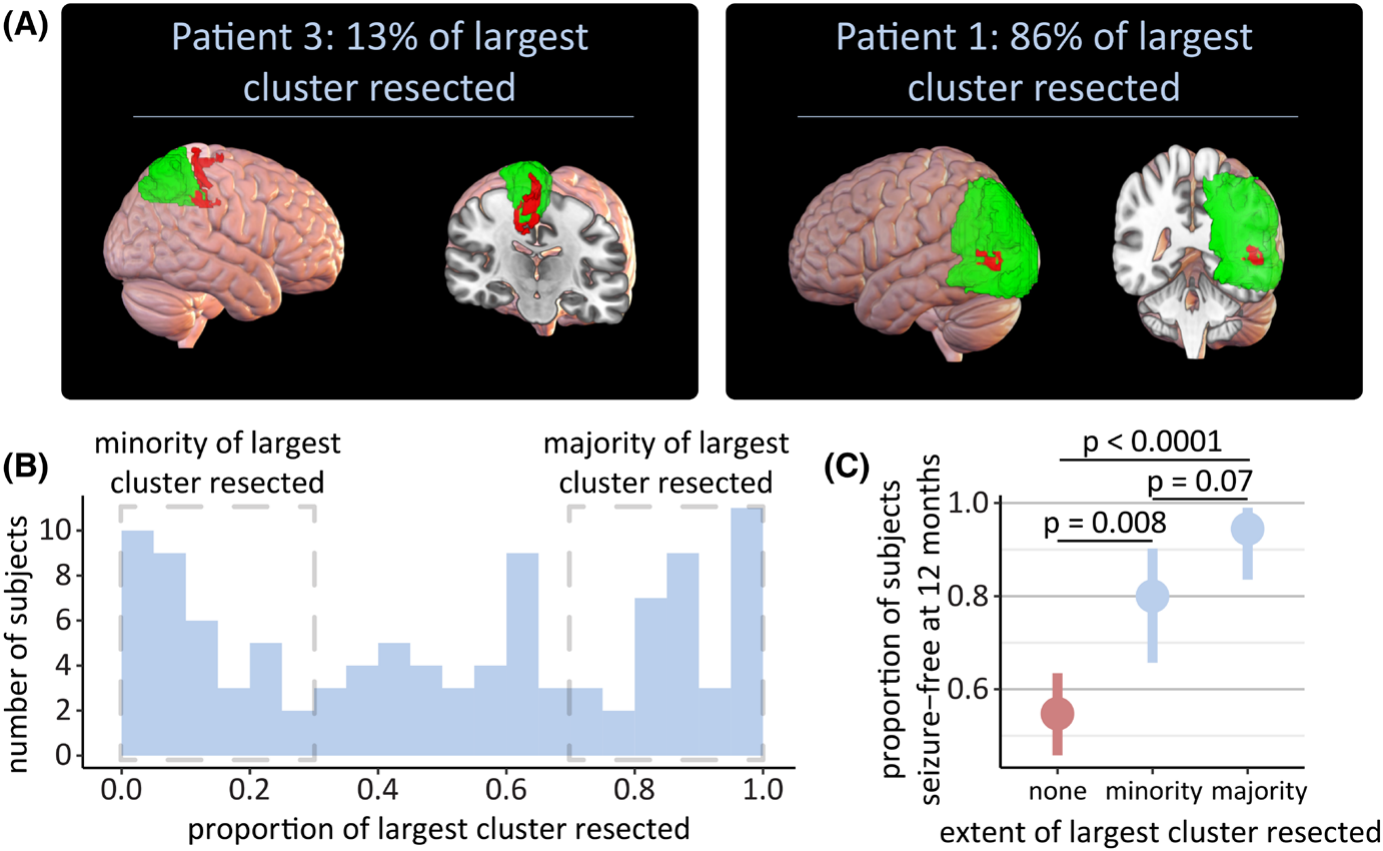
Resection of even a small proportion of the largest cluster can lead to a good outcome. (A) The proportion of the largest cluster resected are shown for two example subjects. Additional example subjects are shown in Supplementary Analysis S10. In the left‐hand subpanel, a small proportion (13%) of the largest cluster was resected. In the right‐hand subpanel, a large proportion (86%) of the largest cluster was resected. (B) Histogram showing the proportion of the largest cluster resected for those subjects with an overlap between the resection and the largest cluster. People who had no more than 30% (small proportion) or more than 70% (large proportion) of the largest cluster resected are identified. (C) Resection of a small (p=.008) and resection of a large proportion (p<.0001) of the largest cluster were both associated with an improved chance of seizure freedom at 1 year postsurgery. Error bars indicate 90% confidence intervals.

In the previous analysis, if the largest cluster overlapped at all with the resection mask, the cluster was classed as resected. However, this did not answer whether the amount of overlap is important in predicting postsurgical seizure freedom. In this analysis, we only considered seizure freedom at 12 months to maximize the sample size. Of the 105 individuals with the largest cluster resected, a wide range in proportion resected was observed (Figure 3B; median = 51%, interquartile range = 65%).

To investigate the importance of the proportion of the largest cluster resected on predicting outcome, we considered two subsets of the data. The first subset considered only those with a small proportion (no more than 30%) of the largest cluster resected (n=35). Of these subjects, 28 (80%) were seizure‐free at 1 year postsurgery. Despite minimal overlap between the largest cluster and the resection, these subjects were still significantly more likely to be seizure‐free, compared to those with no overlap between the largest cluster and the resection (p=.008; Figure 3C).

The second subset considered only those with a large proportion (at least 70%) of the largest cluster resected (n=37). Of these subjects, 34 (94%) were seizure‐free at 1 year postsurgery. These subjects were significantly more likely to be seizure‐free compared to those with no overlap between the largest cluster and the resection (p<.0001; Figure 3C) but not significantly more likely to be seizure‐free than those with a small proportion of the largest cluster resected (p=.07).

### 3.3 Cluster number, volume, and distribution do not explain outcome

**FIGURE 4:**
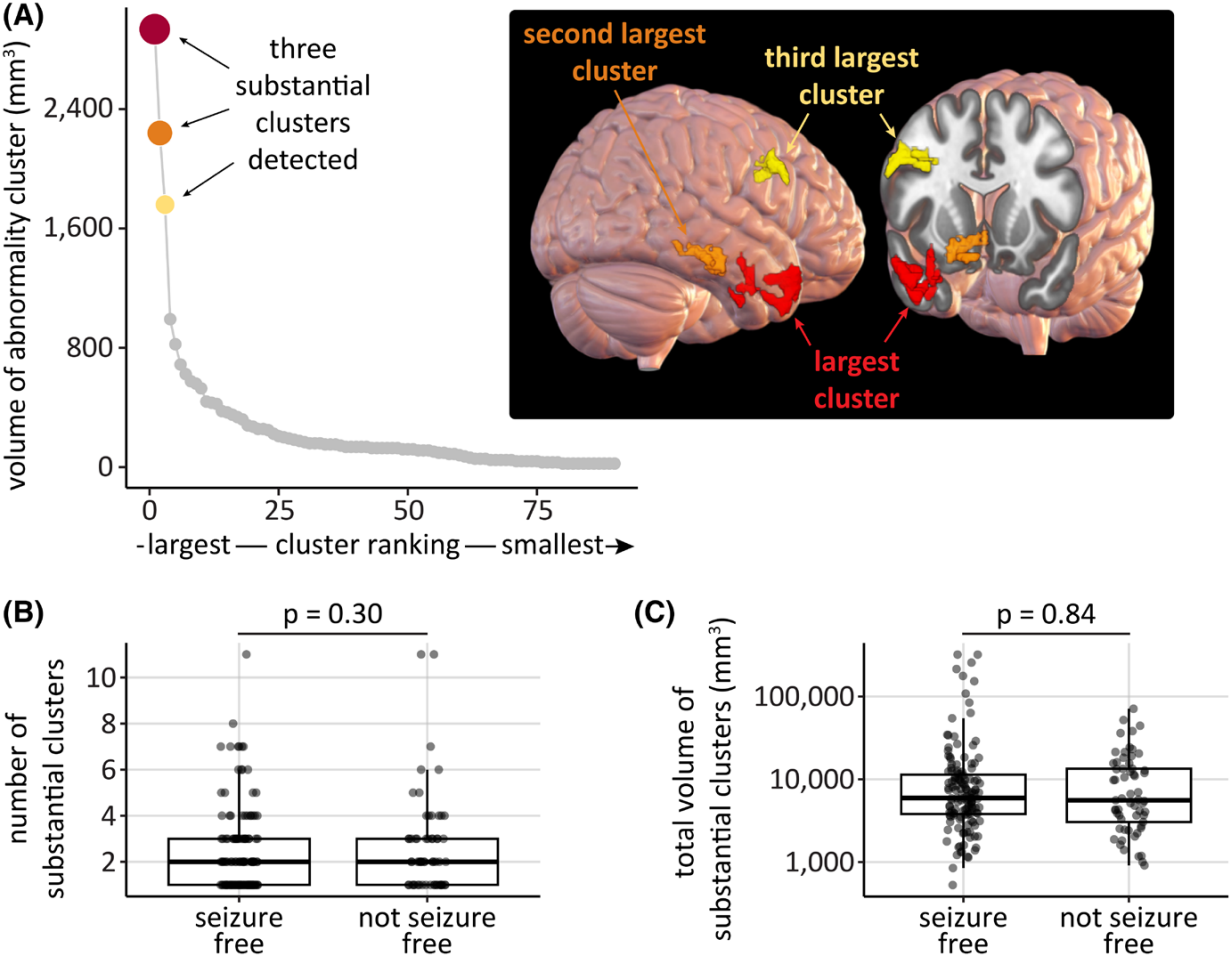
Multiple substantial abnormality clusters may exist, but this does not explain outcome. (A) Within a subject, clusters were ranked by volume. The number of substantial clusters was determined using change‐point analysis. In this example subject, three substantial clusters were detected. (B, C) Neither (B) the number of substantial clusters detected (p=.30) nor (C) the volume of substantial clusters (p=.84) explained outcome at 1 year postsurgery.

More than one abnormal cluster may exist in some patients. Other substantially large clusters were detected using change point analysis (Figure 4A). The number of substantial clusters detected across the cohort was between 1 and 11 (Figure 4B), but this did not differ between seizure‐free and not seizure‐free subjects (p=.30). Similarly, the number of abnormal voxels contained within the substantial clusters did not differ between seizure‐free and not seizure‐free subjects (p=.84).

For those subjects with several clusters detected, the clusters were often in multiple lobes, and no significant differences in the number of lobes affected were observed between seizure‐free and not seizure‐free subjects (Supplementary Analysis S9). Taken together, the number, total volume, and distribution of clusters were not related to outcome.

### 3.4 Resection of other clusters can still lead to a good outcome

**FIGURE 5:**
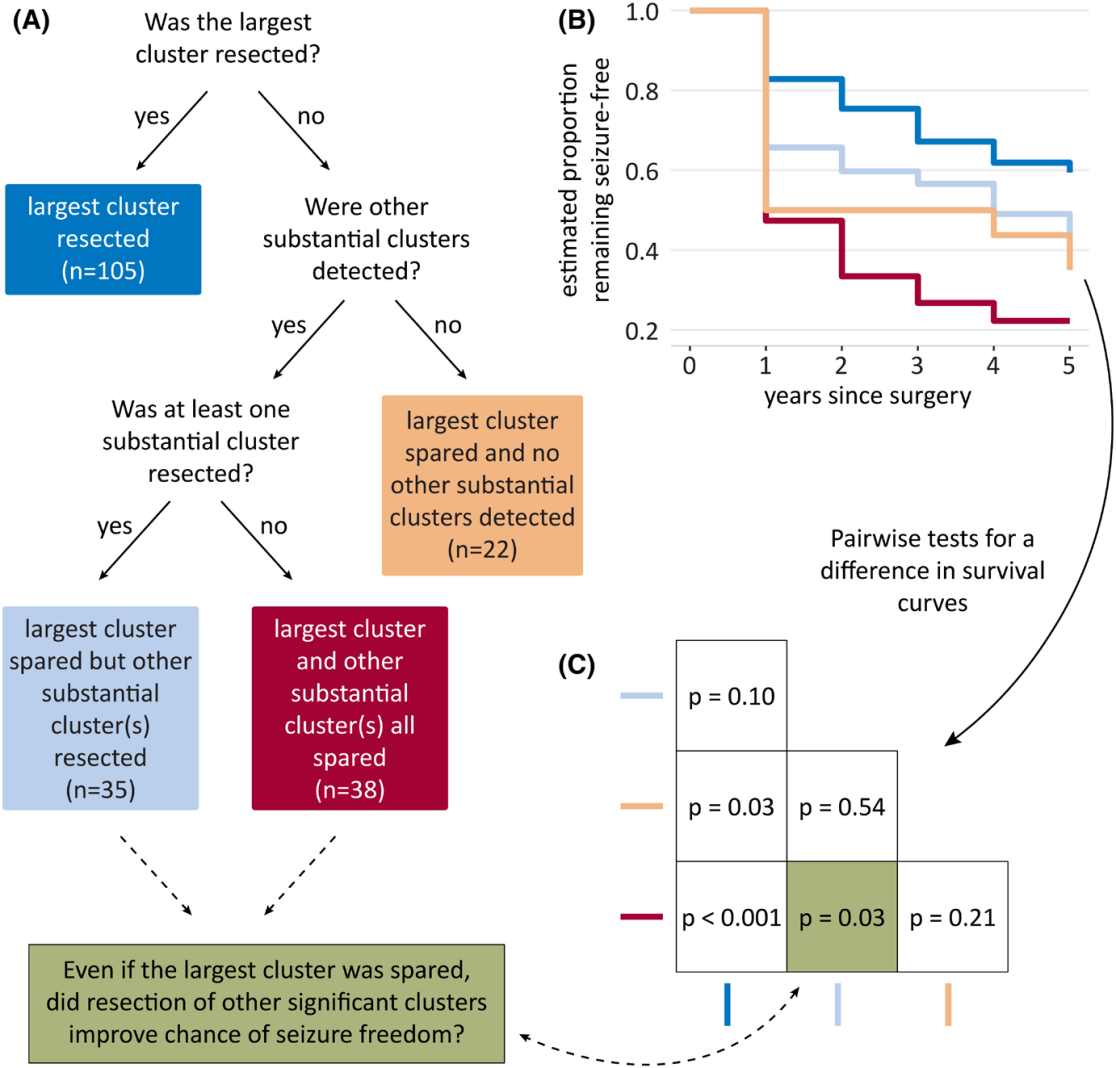
Resection of other substantial clusters may improve seizure freedom. (A) A decision tree was used to investigate whether, if the largest cluster was spared, the resection of other substantial clusters was related to an improved chance of seizure freedom. (B, C) Survival analysis showed that if the largest cluster was spared and other substantial clusters were detected, the resection of these other substantial clusters was related to an improved chance of seizure freedom (p=.03). The best rates of seizure freedom occurred when the largest cluster was resected.

Next, we incorporated information about whether these substantially large clusters were resected into a survival analysis. We were particularly interested in considering the scenario in which the largest cluster was spared (Figure 5A). The possibilities were as follows:

1. Largest cluster was spared and no other substantial clusters were detected (*n* = 22);
2. Largest cluster was spared and at least one other substantial cluster was resected (*n* = 35); and
3. Largest cluster and all other substantial clusters were spared (*n* = 38).

The highest rates of seizure freedom occurred when the largest cluster was resected (83% seizure‐free at 1 year; Figure 5B), and the lowest rates of seizure freedom occurred when multiple substantial clusters existed but none was resected (47% seizure‐free at 1 year). In the case where the largest cluster was spared, but other substantially large clusters existed, resection of at least one of these clusters significantly improved the probability of seizure freedom (p=.03; Figure 5C).

## 4 DISCUSSION

In this work, we present an approach to identifying clusters of abnormalities from diffusion‐weighted MRI. We show that resection of these clusters may lead to an improved likelihood of seizure freedom in people with epilepsy. We demonstrated robustness across scanning type with replication in two independent cohorts.

The detection of epileptogenic abnormalities is crucial to improving rates of seizure freedom after surgery for drug‐resistant focal epilepsy. People may continue to have seizures after surgery for several reasons, including (1) if the correct part of the brain was targeted but resection size was insufficient or (2) the wrong part of the brain was targeted or there are multifocal epileptogenic regions. Our method detects abnormalities in individual voxels, which may delineate (1) the extent and (2) the location of the EZ. It is particularly important to develop methods that can localize the EZ in cases with no visual abnormalities, because these people also have lower rates of seizure freedom.^33^ Uncertain localization of the EZ is also the main reason for not having potentially curative epilepsy surgery.^34^ Our approach may be valuable for those subjects who were clinically assessed as MRI‐negative (Supplementary Analysis S4), although the smaller sample size meant that this did not reach statistical significance, despite the similar effect size. Another benefit of our approach is the potential impact on minimally invasive surgeries. If the suspected epileptogenic tissue can be clearly delineated with high precision, then the removal of adjacent healthy brain tissue can be minimized. Our mapping of abnormalities in individuals at the spatial resolution of individual voxels could, in the future, have applications for more localized treatment techniques, as the field explores minimally invasive approaches beyond resective surgery.^35^ Regardless of the eventual treatment option, the clear identification of brain abnormalities is of huge clinical importance.

We showed that resection of the largest cluster was associated with an improved likelihood of seizure freedom, where resection was defined as any overlap between the largest cluster and the resection mask. Importantly, we also demonstrated that complete resection of the largest cluster was not required for an improved rate of seizure freedom. This may be important if all of the largest cluster cannot be resected due to proximity to eloquent cortex (e.g., Patient 3 in Figure 3A). The risk of a significant deficit resulting from a resection in brain areas associated with motor or language function is another common reason for not having epilepsy surgery.^34^ Resecting only the part of the largest cluster not in eloquent cortex may therefore optimize the likelihood of seizure freedom, while minimizing the risk to crucial functions.

Some people achieved seizure freedom after surgery, despite not having the largest (or any substantial) cluster resected. Diffusion abnormalities and epileptogenic tissue are different concepts, and it is evident that not all areas with abnormal dwMRI signal are epileptogenic. Nevertheless, biomarkers with high sensitivity (but lower specificity) may still be useful in practice, especially when used in parallel with other modalities. There were people with abnormal clusters in multiple lobes that were not all resected (Figure 4B) and who became seizure‐free. In addition, epilepsy is a network disorder,^36^ and epilepsy surgery has a significant impact on the wider structural connectome.^37^ The disconnection of critical white matter tracts may have widespread effects. Resection may have sufficient impact on the epileptogenic network to prevent further seizures even if the largest (or any substantial) cluster was not resected. Future work could extend our analyses to consider the hubness of individual voxels, in addition to the abnormality, to provide additional insight,^38^ because network properties have previously been shown to relate to outcome.^9^, ^10^, ^37^, ^39^

MD quantifies the average diffusion in all directions in each voxel in the brain. Unrestricted diffusion, as seen in free water, results in higher MD values than in tightly packed neurons. Increased white matter MD is often reported in epilepsy^4^ and is thought to reflect myelin disruption and increased extracellular space.^40^ Our results are similarly driven by MD increases (Supplementary Analysis S11). In our main analysis, we do not restrict ourselves to solely white matter voxels, because gray matter is clearly crucial in epilepsy (see Supplementary Analysis S12 for white matter only analysis).^41^ Gray matter diffusion abnormalities may represent a breakdown in cellular microstructure and have been investigated for their use as biomarkers in other neurological disorders.^42^ Future work will investigate the relative predictive ability of abnormality clusters derived from multicompartment metrics, which may relate more directly to underlying tissue microstructure than the traditional diffusion tensor metrics used in this study.^13^, ^43^ Although testing whether our findings replicate studies using other imaging sequences (e.g., T1‐weighted MRI or fluid‐attenuated inversion recovery) is beyond the scope of this work, it remains a clinically relevant avenue for future investigation.

This study has limitations. The abnormalities that we calculate do not account for age and sex due to the computational and technical infeasibility of regressing out these covariates in every voxel. Our results are promising, given the relationship between diffusion tensor values and both age and sex in health.^44^ New methods to account for these covariates should only improve the accuracy of calculated abnormalities. In addition, abnormalities were computed by comparison to two relatively small cohorts of controls. To further strengthen this work in the future, normative models of diffusion‐weighted MRI should be developed, as they have in other modalities.^45^, ^46^, ^47^, ^48^ These normative models should be trained on a large number of controls and can act as a comprehensive healthy baseline against which abnormalities can be calculated.^49^

## 5 CONCLUSIONS

In summary, we present an approach to identify focal brain abnormalities from dwMRI. We show retrospectively that, following epilepsy surgery, the likelihood of seizure freedom may be improved by the resection of dwMRI abnormalities. Thus, dwMRI analysis may offer a useful tool to help localize the epileptogenic region(s) and guide the location and extent of resection to improve surgical outcomes.

## AUTHOR CONTRIBUTIONS

J.H. and P.N.T. contributed to the conception and design of the study. All authors contributed to the acquisition and analysis of data. J.H. and P.N.T. contributed to drafting the text or preparing the figures.

## CONFLICT OF INTEREST STATEMENT

None of the authors has any conflict of interest to disclose. We confirm that we have read the Journal's position on issues involved in ethical publication and affirm that this report is consistent with those guidelines.

## Supporting information


Table S1.

## Data Availability

The data that support the findings of this study are available from the corresponding author upon reasonable request.
