# Supplementary material for: Seizure freedom after surgical resection of diffusion‐weighted magnetic resonance imaging abnormalities
Source: Epilepsia. 2025 Jun 10;66(9):3480–90. doi: 10.1111/epi.18490 (PMC12455403; doi:10.1111/epi.18490)
Supplement: Supplementary file 1 — Table S1. [file EPI-66-3480-s001.docx]

**Supplementary Contents**

**Supplementary Table 1 - Patient cohort data**

**Supplementary Table 2 - Sample sizes associated with Figure 2**

**Supplementary Analysis 1 - Alternative thresholds**

**Supplementary Analysis 2 - FA, RD and AD abnormalities**

**Supplementary Analysis 3 - Comparison of predictive ability of the largest cluster vs clinical features**

**Supplementary Analysis 4 - MRI-positive and MRI-negative subgroup analysis**

**Supplementary Analysis 5 - Scanning protocol subgroup analysis**

**Supplementary Analysis 6 - TLE and ETLE subgroup analysis**

**Supplementary Analysis 7 - Right and left hemisphere resection subgroup analysis**

**Supplementary Analysis 8 - Resection size analysis**

**Supplementary Analysis 9 - Lobes affected by substantial abnormality clusters**

**Supplementary Analysis 10 - Additional subject brain plots**

**Supplementary Analysis 11 - MD increases only**

**Supplementary Analysis 12 - WM voxels only**

**Supplementary Table 1 - Patient cohort data**

|  | **ILAE 1,2** | **ILAE 3+** | **Test statistic** |
| --- | --- | --- | --- |
| **n** | 139 | 61 |  |
| **Onset age, median (IQR)** | 12 (14.5) | 15 (13.0) | W = 3617, p = 0.10 |
| **Sex, male:female** | 56:83 | 33:28 | 𝛸^2^ = 2.74, p = 0.10 |
| **Type, temporal:extratemporal** | 108:31 | 47:14 | 𝛸^2^ ≈ 0.00, p = 1 |
| **Side, left:right** | 80:59 | 24:37 | 𝛸^2^ = 4.93, p = 0.03 |
| **MRI, non-lesional:lesional** | 18:121 | 14:47 | 𝛸^2^ = 2.45, p = 0.12 |

**Table S1: Patient data by 12 month post-surgical seizure freedom.** The difference in onset age between groups was assessed using a Wilcoxon rank-sum test. Other differences between groups were assessed using Chi-squared tests.

**Supplementary Table 2 - Sample sizes associated with Figure 2**

| At Risk |  | **Years since surgery** | | | | | |
| --- | --- | --- | --- | --- | --- | --- | --- |
|  |  | **0** | **1** | **2** | **3** | **4** | **5** |
| **Largest cluster** | **Resected** | 105 | 105 | 78 | 55 | 38 | 25 |
|  | **Spared** | 95 | 95 | 50 | 39 | 29 | 18 |

| Event* |  | **Years since surgery** | | | | | |
| --- | --- | --- | --- | --- | --- | --- | --- |
|  |  | **0** | **1** | **2** | **3** | **4** | **5** |
| **Largest cluster** | **Resected** | 0 | 18 | 25 | 31 | 34 | 35 |
|  | **Spared** | 0 | 43 | 50 | 53 | 57 | 59 |
| * event = cumulative seizure recurrence since surgery | | | | | | | |

**Table S2: Sample sizes corresponding to Figure 2.**

**Supplementary Analysis 1 - Alternative thresholds**

In the main text, we presented results which classified voxels as abnormal if they exceed the z-score threshold of 3. Here, we replicated the main result finding with alternative thresholds (Supplementary Figure 1). Specifically, we found that an overlap between the largest abnormal cluster and the resection was associated with an increased rate of seizure freedom over five years using alternative threshold values of 6 (p=0.007), 5 (p=0.005), 4 (p<0.001) and 2 (p=0.2). These results suggest that our approach is relatively robust to the choice of threshold for classifying voxels as abnormal.


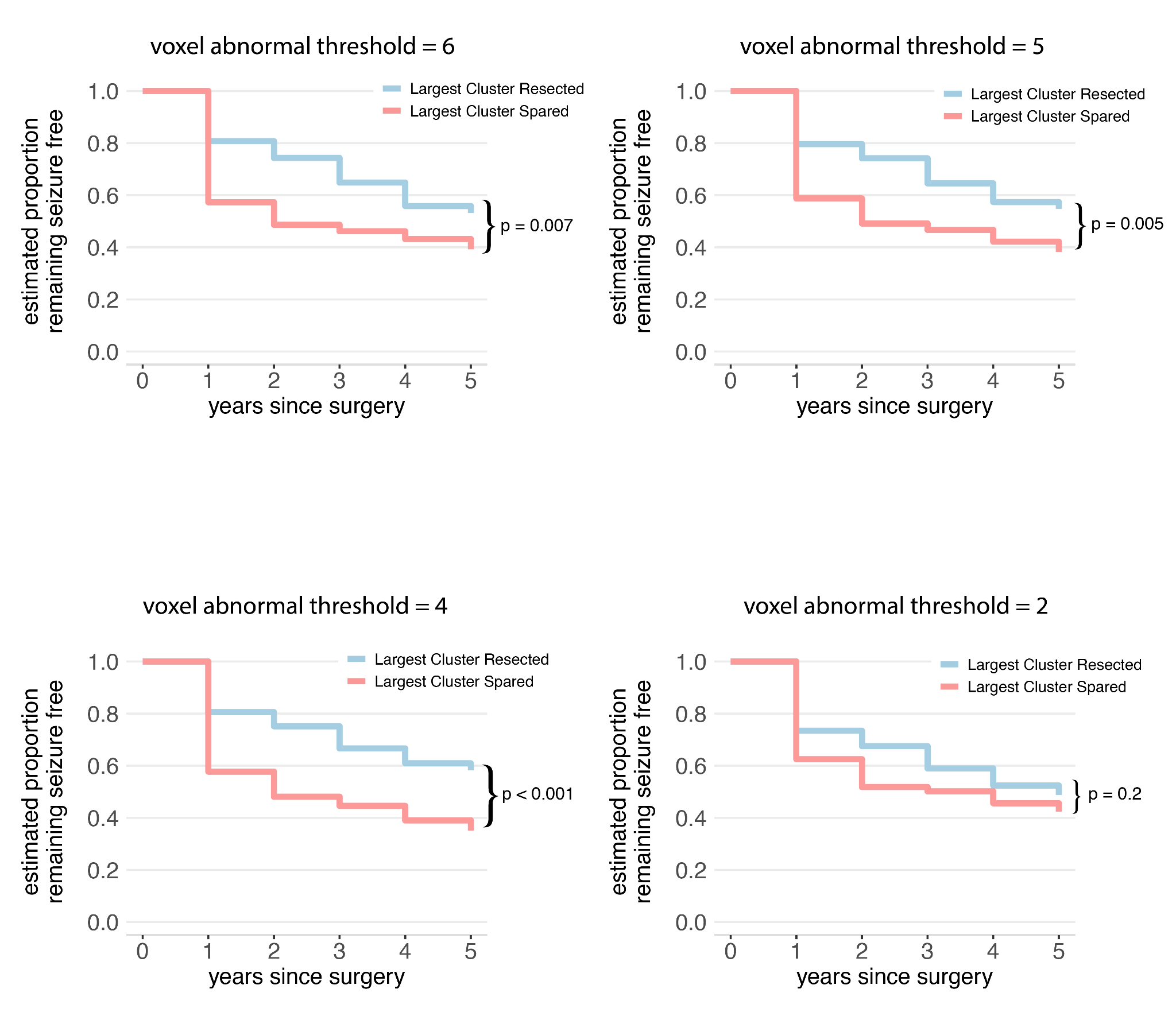


**Supplementary Figure 1**: Resecting the largest abnormal cluster was associated with an improved likelihood of seizure freedom using alternative voxel abnormality thresholds of 6 (top-left; p = 0.007), 5 (top-right; p = 0.005), 4 (bottom-left; p < 0.001) and 2 (bottom-right; p = 0.2).

**Supplementary Analysis 2 - FA, RD and AD abnormalities**

In the main text, we presented results using abnormalities derived from mean diffusivity (MD). Here, we replicated the main result using different diffusion metrics (Supplementary Figure 2). We found similar results - that an overlap between the largest abnormal cluster and the resection was associated with an increased rate of seizure freedom over five years using FA (p=0.06), RD (p=0.01) and AD (p=0.002).


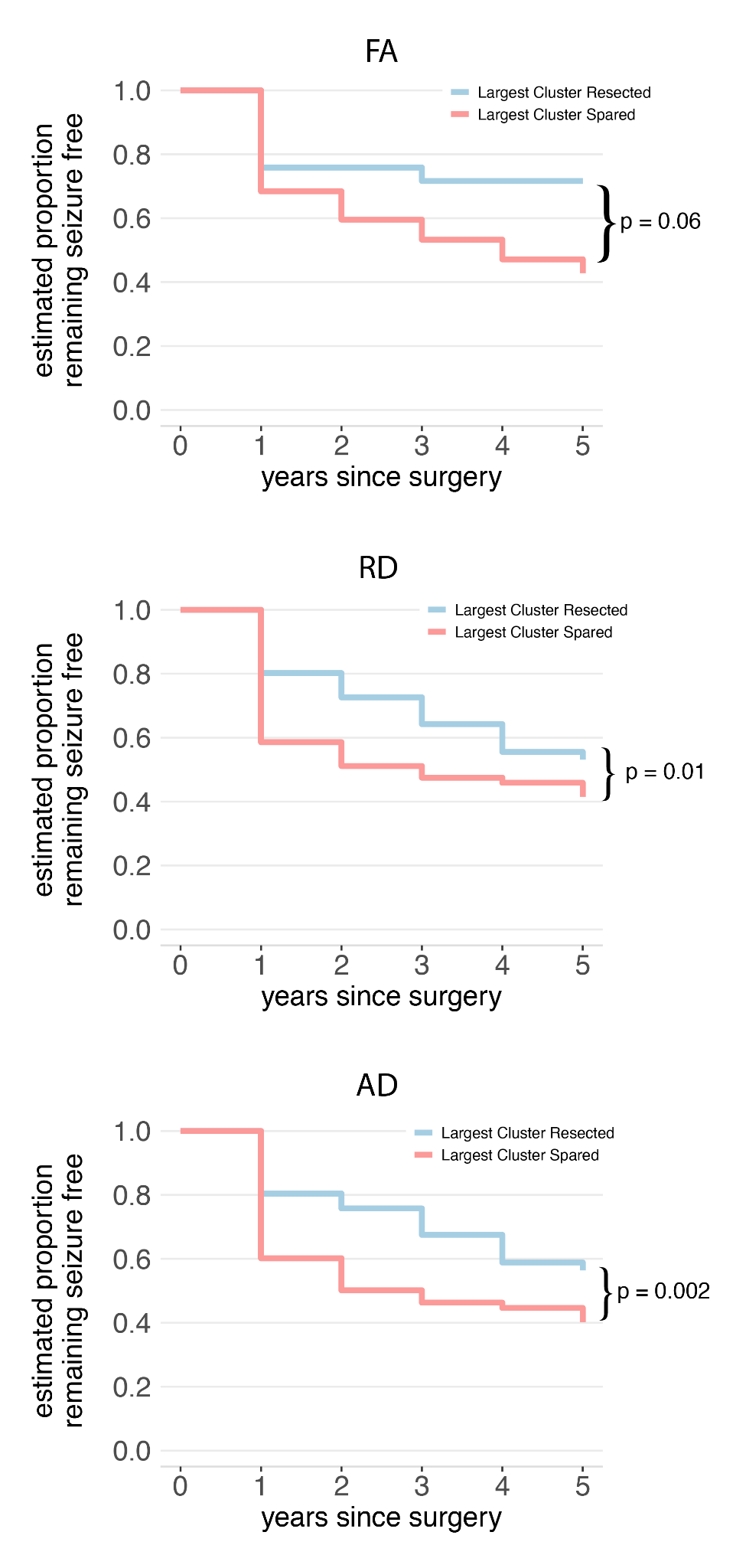


**Supplementary Figure 2**: Resecting the largest abnormal cluster was associated with an improved likelihood of seizure freedom using FA (top; p = 0.06), RD (middle; p = 0.01) and AD (bottom; p = 0.002).

**FA**

| At Risk |  | **Years since surgery** | | | | | |
| --- | --- | --- | --- | --- | --- | --- | --- |
|  |  | **0** | **1** | **2** | **3** | **4** | **5** |
| **Largest cluster** | **Resected** | 26 | 26 | 19 | 16 | 13 | 10 |
|  | **Spared** | 174 | 174 | 109 | 78 | 54 | 33 |

| Event* |  | **Years since surgery** | | | | | |
| --- | --- | --- | --- | --- | --- | --- | --- |
|  |  | **0** | **1** | **2** | **3** | **4** | **5** |
| **Largest cluster** | **Resected** | 0 | 6 | 7 | 8 | 8 | 8 |
|  | **Spared** | 0 | 55 | 68 | 76 | 83 | 86 |
| * event = cumulative seizure recurrence since surgery | | | | | | | |

**RD**

| At Risk |  | **Years since surgery** | | | | | |
| --- | --- | --- | --- | --- | --- | --- | --- |
|  |  | **0** | **1** | **2** | **3** | **4** | **5** |
| **Largest cluster** | **Resected** | 101 | 101 | 74 | 53 | 37 | 23 |
|  | **Spared** | 99 | 99 | 54 | 41 | 30 | 20 |

| Event* |  | **Years since surgery** | | | | | |
| --- | --- | --- | --- | --- | --- | --- | --- |
|  |  | **0** | **1** | **2** | **3** | **4** | **5** |
| **Largest cluster** | **Resected** | 0 | 19 | 26 | 33 | 38 | 39 |
|  | **Spared** | 0 | 42 | 49 | 51 | 53 | 55 |
| * event = cumulative seizure recurrence since surgery | | | | | | | |

**AD**

| At Risk |  | **Years since surgery** | | | | | |
| --- | --- | --- | --- | --- | --- | --- | --- |
|  |  | **0** | **1** | **2** | **3** | **4** | **5** |
| **Largest cluster** | **Resected** | 93 | 93 | 70 | 56 | 39 | 24 |
|  | **Spared** | 107 | 107 | 58 | 38 | 28 | 19 |

| Event* |  | **Years since surgery** | | | | | |
| --- | --- | --- | --- | --- | --- | --- | --- |
|  |  | **0** | **1** | **2** | **3** | **4** | **5** |
| **Largest cluster** | **Resected** | 0 | 17 | 21 | 28 | 33 | 34 |
|  | **Spared** | 0 | 44 | 54 | 56 | 58 | 60 |
| * event = cumulative seizure recurrence since surgery | | | | | | | |

**Table S2: Sample sizes corresponding to Supplementary Figure 2.**

**Supplementary Analysis 3 - Comparison of predictive ability of the largest cluster vs clinical features**

Post-surgical outcomes may be influenced by various clinical factors, such as epilepsy duration. We compared the ability of resecting the largest abnormal cluster to predict seizure outcomes against the eight clinical features for which data were available to us: side of resection, sex, age at epilepsy onset, history of status epilepticus, epilepsy duration, presence of a lesion on MRI (MRI-negative or positive), number of anti-seizure medications (ASMs) tried before surgery, and history of focal to bilateral tonic-clonic seizures (FBTCS). This approach and clinical features are similar to previous work (Sinha et al. 2021, <https://doi.org/10.1212/WNL.0000000000011315>).

We used a logistic regression model to distinguish seizure freedom (ILAE 1 or 2 vs ILAE 3), incorporating each of the nine predictors. For each model size, we applied best subset selection using the R *leaps* package to identify the optimal combination of predictors. For instance, in a model of size five, only the five variables contributing to the best model fit (lowest AIC) were included.

To assess feature importance, we normalised the absolute (standardised) coefficients of each variable, ensuring the most important feature had a value of one. Whether the largest cluster was resected was the most informative predictor for each model size (Supplementary Figure 11).

**
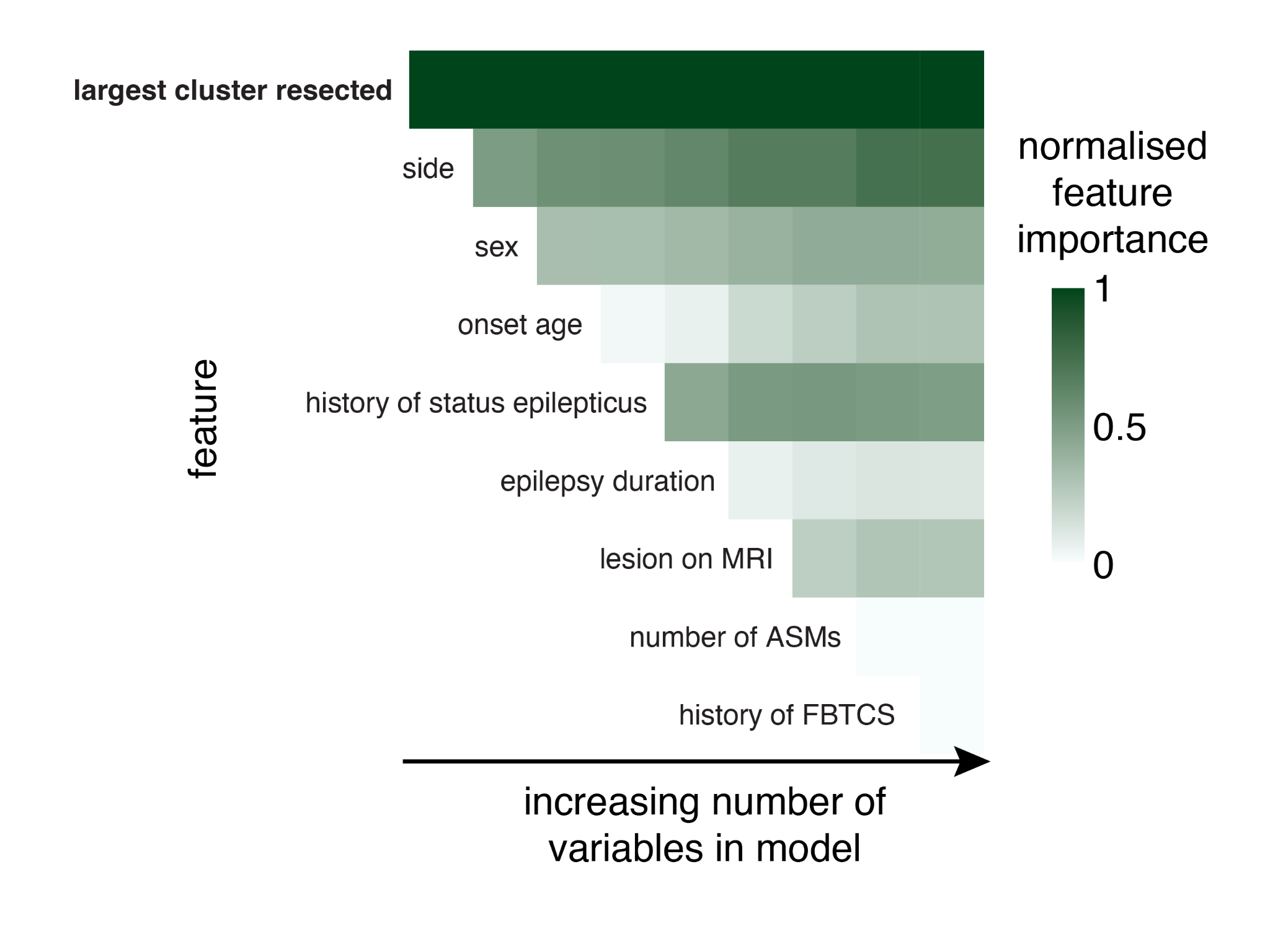
**

**Supplementary Figure 3**: Resecting the largest abnormal cluster was more informative in distinguishing post-surgical outcome groups than clinical variables.

**Supplementary Analysis 4 - MRI-positive and MRI-negative subgroup analysis**

In the main text, we presented results for the full cohort. However, dMRI may be of particular use clinically where existing (e.g. T1w) MRI sequences are unable to detect any abnormality (i.e. MRI-negative). As a result, we tested our approach separately on clinically-marked MRI-positive (n=168) and MRI-negative (n=32) subgroups (Supplementary Figure 3). We found that the same pattern was observed in both subgroups separately, with an increased likelihood of seizure freedom if the largest cluster was resected (left: MRI-negative, p=0.07; right: MRI-positive, p=0.002). These results suggest that our approach is effective at delineating epileptogenic tissue in both clinically marked MRI-negative and MRI-positive cases.


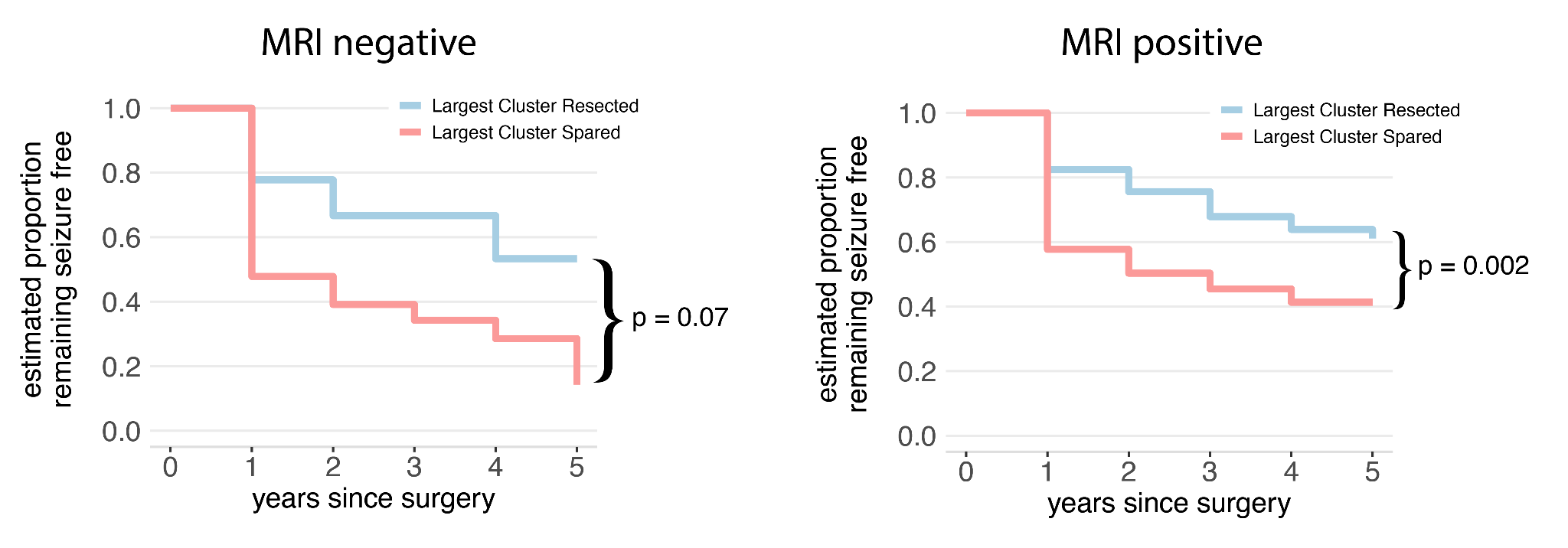


**Supplementary Figure 4**: Resecting the largest abnormal cluster was associated with an improved likelihood of seizure freedom in both MRI-negative (left; p = 0.07) and MRI-positive (right; p = 0.002) subgroups.

**Supplementary Analysis 5 - Scanning protocol subgroup analysis**

In the main text, we presented results for the full cohort. However, this cohort was obtained as part of two separate subgroups using different scanning protocols. The first cohort was collected between 2009 and 2013, and had 107 patients and 29 controls. The second cohort was collected between 2014 and 2019, and had 93 patients and 68 controls. We tested our approach separately on both the subgroups (Supplementary Figure 4). We found that the same pattern was observed in both subgroups separately, with an increased likelihood of seizure freedom if the largest cluster was resected (left: first cohort, p=0.03; right: second cohort, p < 0.001). These results suggest that our approach is effective at delineating epileptogenic tissue even when using only a small number (n=29) of controls as a baseline, and using older scanning protocols.


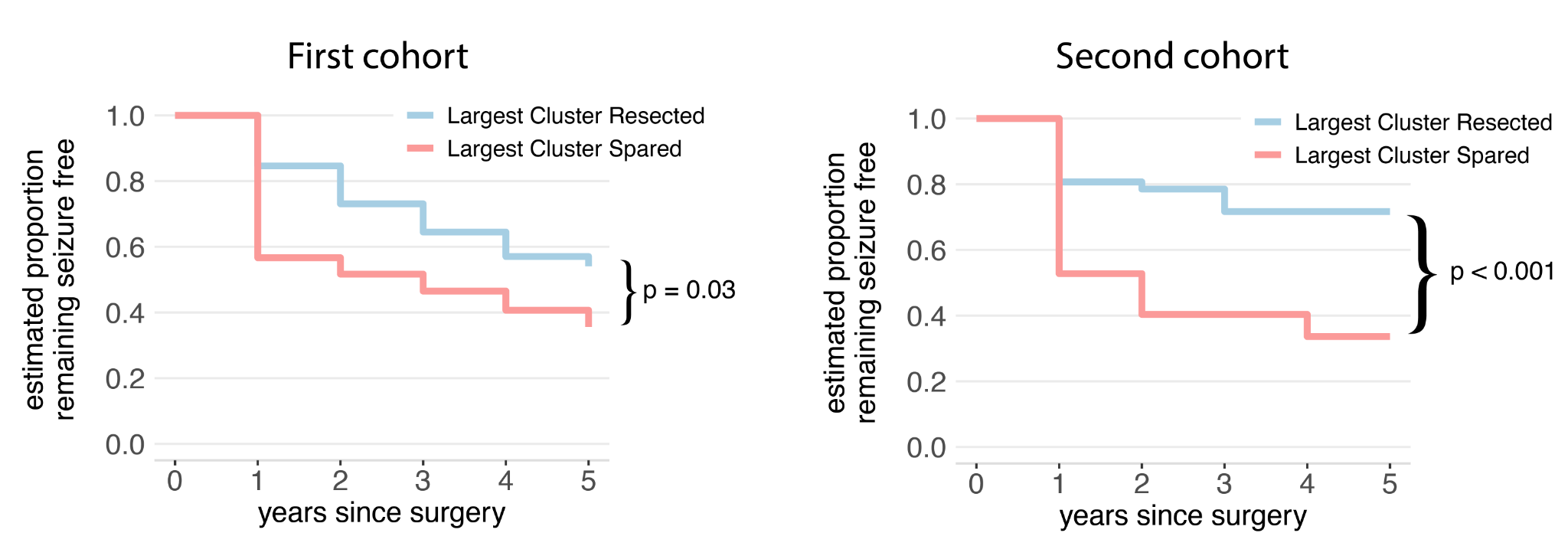


**Supplementary Figure 5**: Resecting the largest abnormal cluster was associated with an improved likelihood of seizure freedom in both scanning protocol subgroups (left: first cohort, p=0.03; right: second cohort, p<0.001).

**Supplementary Analysis 6 - TLE and ETLE subgroup analysis**

In the main text, we presented results for the full cohort. This cohort comprised both TLE (n=156) and ETLE (n=44) subjects. We tested our approach separately on both TLE and ETLE (Supplementary Figure 5). We found that the same pattern was observed in both subgroups separately, with an increased likelihood of seizure freedom if the largest cluster was resected (left: TLE, p=0.0003; right: ETLE, p = 0.10). These results suggest that our approach is effective at delineating epileptogenic tissue on both TLE and ETLE subjects.


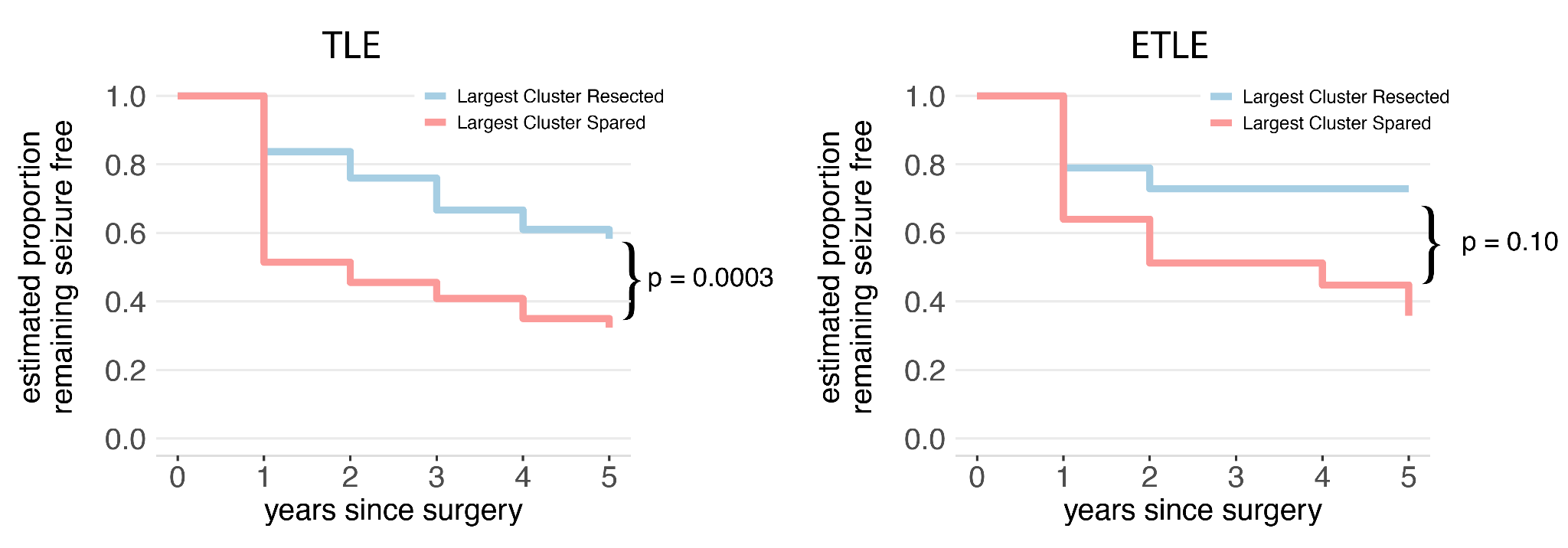


**Supplementary Figure 6**: Resecting the largest abnormal cluster was associated with an improved likelihood of seizure freedom in both TLE (left, p=0.0003) and ETLE (right, p=0.10).

**Supplementary Analysis 7 - Right and left hemisphere resection subgroup analysis**

In the main text, we presented results for the full cohort. This cohort comprised of subjects with both left (n=104) and right (n=96) hemisphere resections. We tested our approach separately on both left and right hemisphere resections (Supplementary Figure 6). We found that the same pattern was observed in both subgroups separately, with an increased likelihood of seizure freedom if the largest cluster was resected (left: left-sided resection, p=0.002; right: right-sided resection, p = 0.01). These results suggest that our approach is robust to the side of surgical resection.


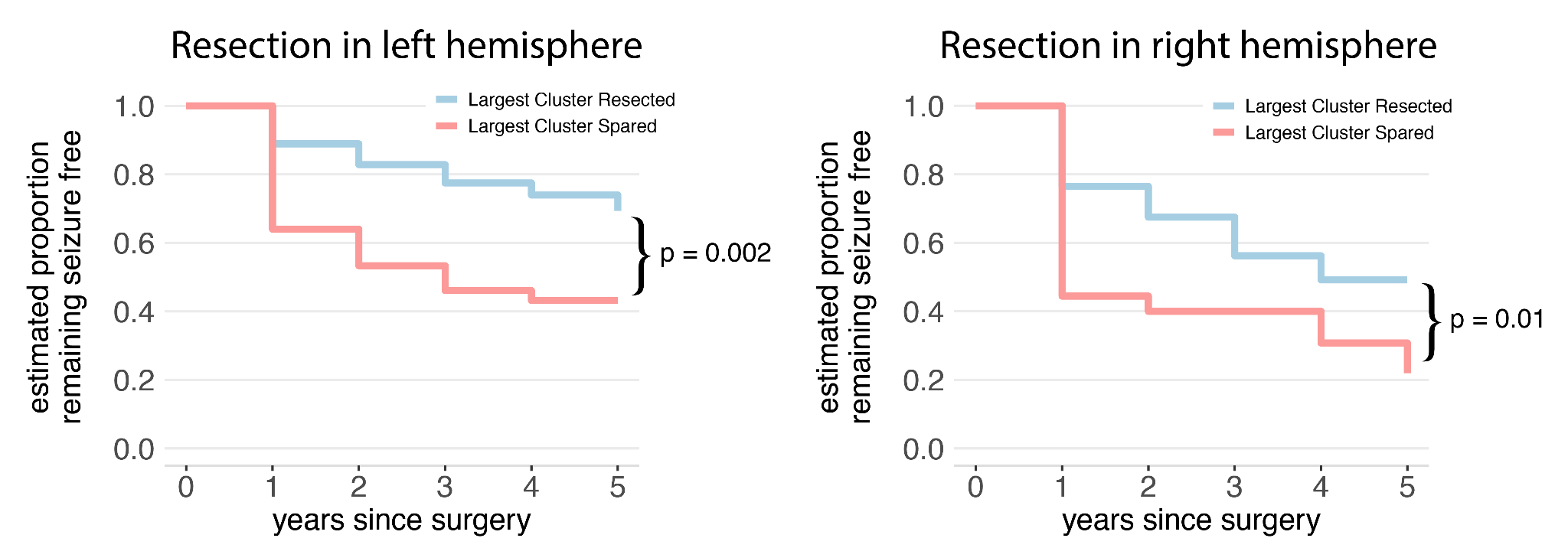


**Supplementary Figure 7**: Resecting the largest abnormal cluster was associated with an improved likelihood of seizure freedom in both left-sided resections (left, p=0.002) and right-sided resections (right, p=0.01).

**Supplementary Analysis 8 - Resection size analysis**

The size of the resection may be an underlying confounder for the relationship between the resection of the abnormal clusters and post-surgical outcome. For bigger resections, the likelihood of containing one of more clusters is much higher, so the effect may simply come from the resection extent itself, not the detected clusters. Therefore, it is crucial to assess whether our findings are influenced by resection size. We compared resection sizes between seizure-free (ILAE 1 and 2) and not seizure-free (ILAE 3+) groups using the Wilcoxon rank-sum test. The analysis revealed no significant difference in resection size between outcome groups (p = 0.16, Supplementary Figure 8). This suggests that it is not the size of the resection, but whether or not it contains abnormal clusters, that is important for distinguishing post-surgical outcome.

**
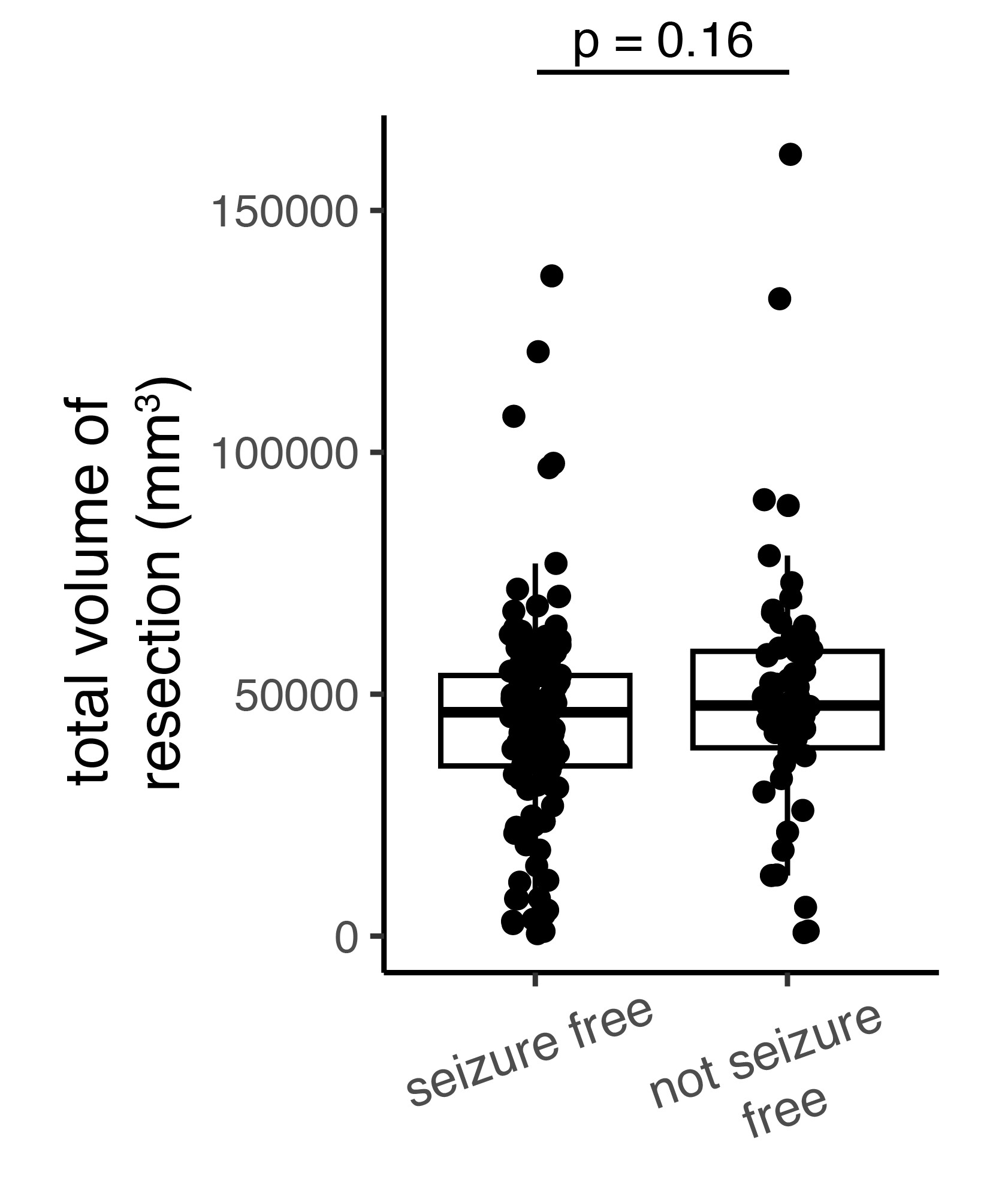
**

**Supplementary Figure 8**: Resection size did not distinguish post-surgical seizure freedom (p=0.16).

**Supplementary Analysis 9 - Lobes affected by substantial abnormality clusters**

In the main text, we did not consider the spatial distribution of abnormal clusters - only whether they were resected or not. In this analysis, we considered how many unique lobes contained an abnormal cluster and investigated whether this differed according to post-surgical outcome (Supplementary Figure 4). Voxels were assigned to regions of interest (ROI) using the Desikan-Killiany standard space atlas, where each ROI was assigned to a lobe. Lobes were classified as frontal, temporal, parietal and occipital on both hemispheres, giving eight lobes in total per subject. We used Fisher’s Exact Test to evaluate whether the number of lobes containing abnormal clusters differed between good (ILAE 1 or 2) and poor (ILAE 3+) outcome subjects.

For those subjects with two substantially abnormal clusters identified, they most often occurred in two unique lobes. The number of lobes the abnormal clusters occurred in did not differ between seizure-free and not-seizure-free subjects (p=0.26).

For those subjects with three substantially abnormal clusters identified, they most often occurred in three unique lobes. The number of lobes the abnormal clusters occurred in did not differ between seizure-free and not-seizure-free subjects (p=0.17).

For those subjects with four substantially abnormal clusters identified, they most often occurred in three unique lobes. The number of lobes the abnormal clusters occurred in did not differ between seizure-free and not-seizure-free subjects (p=0.11).

Taken together, this suggests that the spatial distribution of clusters across lobes did not explain outcome when not considering the location of the resection.


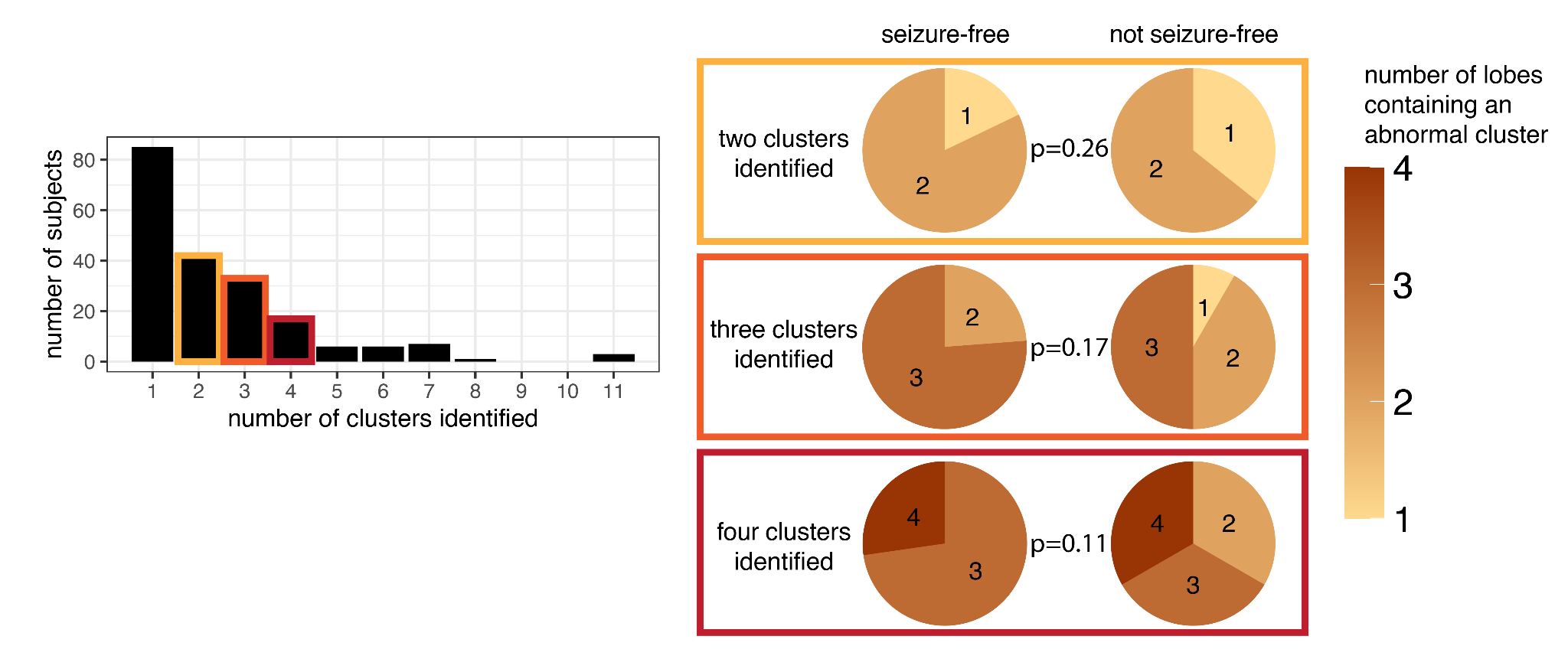


**Supplementary Figure 9**: The number of lobes containing a substantially abnormal cluster did not distinguish post-surgical outcome, when two (p=0.26), three (p=0.17) or four (p=0.11) of these clusters were identified.

**Supplementary Analysis 10 - Additional subject brain plots**

Here, we provide additional examples of subjects who had varying levels of overlap between the largest abnormal cluster and the resection, as a supplement to Figure 3 in the main text. All six subjects were seizure-free (ILAE 1) at 12 months post-surgery. Patient 7 was clinically assessed as MR-negative.


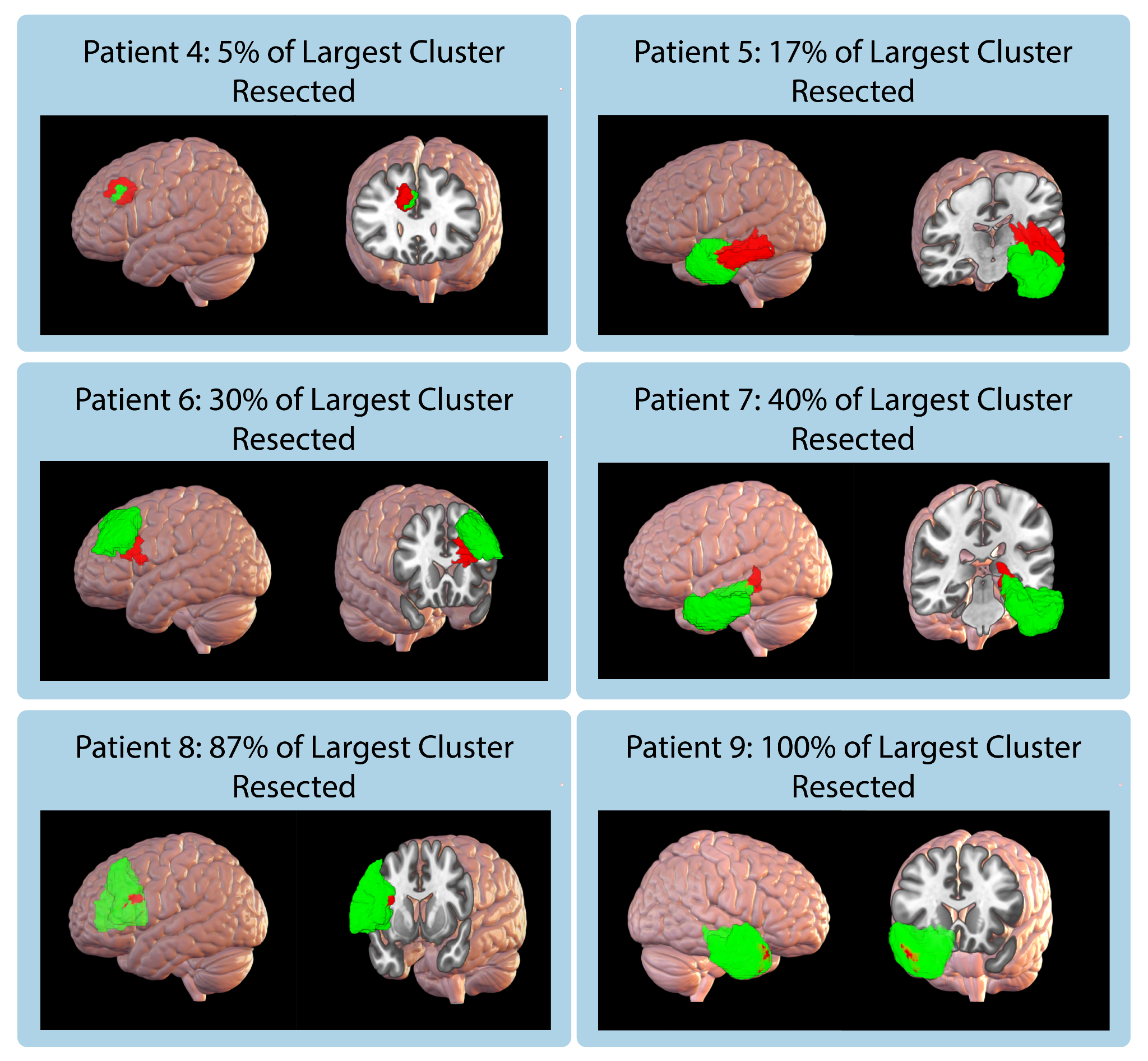


**Supplementary Figure 10**: Six further example subjects are shown with varying proportions of their largest clusters resected. All six subjects were seizure-free (ILAE 1 at 12 months).

**Supplementary Analysis 11 - MD increases only**

In the main text, we presented results which classified voxels as abnormal if they exceed the MD (absolute) z-score threshold of 3. These abnormalities could be either increases or decreases in MD. However, MD increases are typically reported in epilepsy. As a result, we re-ran our analysis, considering voxels as abnormal only if they were positively greater than a z-score of 3. We found that an overlap between the largest abnormal cluster and the resection was associated with an increased rate of seizure freedom over five years (p=0.01). This suggests that it is MD increases that are driving the results reported in the main text.


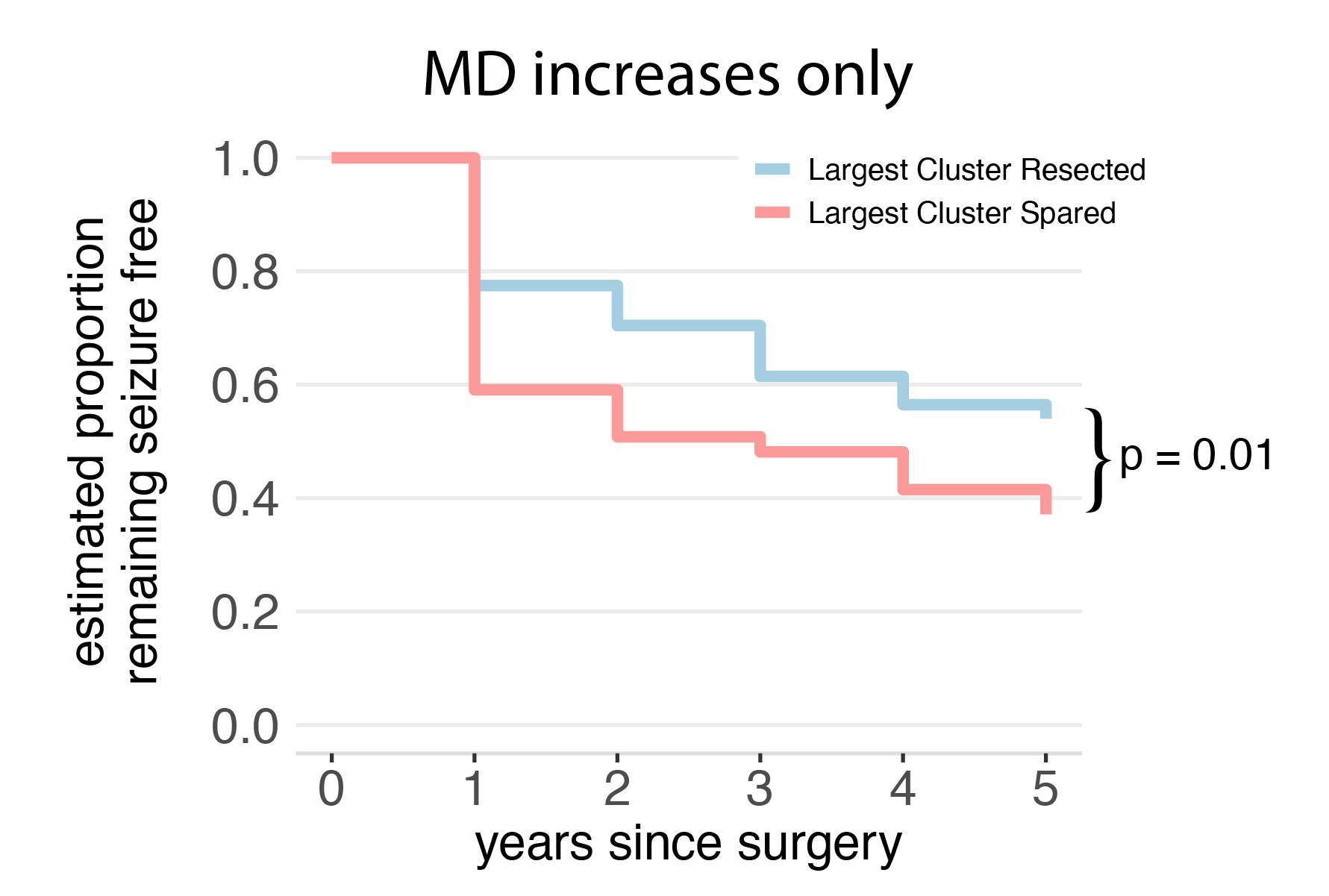


**Supplementary Figure 11**: Resecting the largest abnormal cluster, derived from MD increases only, was associated with an improved likelihood of seizure freedom (p=0.01).

**Supplementary Analysis 12 - WM voxels only**

In the main text, we presented results which classified voxels as abnormal if they exceed the MD z-score threshold of 3. These voxels could be either in the grey matter (GM) or white matter (WM). However, diffusion metric changes are typically reported in white matter in epilepsy. As a result, we re-ran our analysis, to consider only voxels in the white matter. We found that an overlap between the largest abnormal cluster and the resection was associated with an increased rate of seizure freedom over five years (p=0.008).


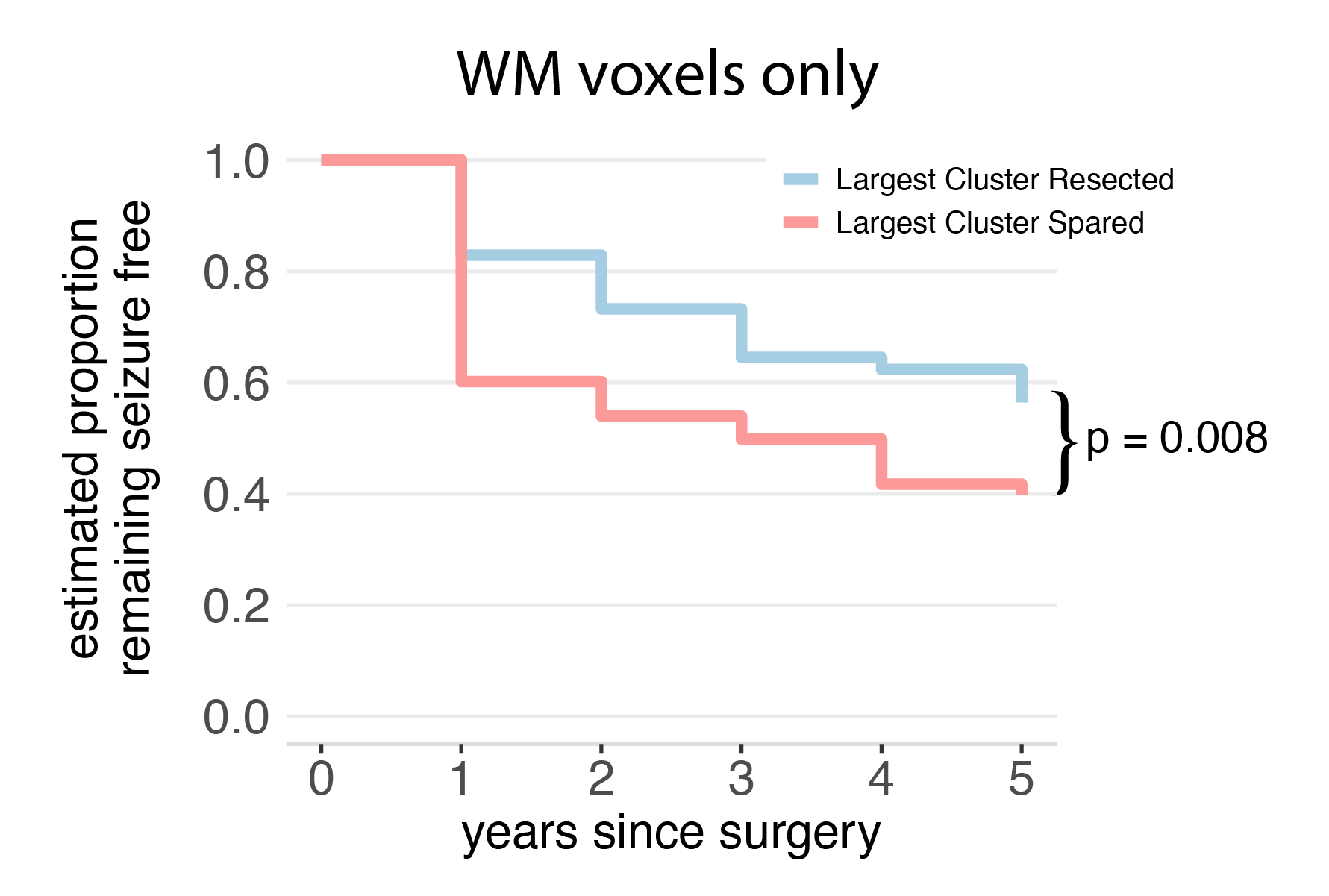


**Supplementary Figure 12**: Resecting the largest abnormal cluster, derived from MD white matter voxel abnormalities only, was associated with an improved likelihood of seizure freedom (p=0.008).
